# Scoutknife: A naïve, whole genome informed phylogenetic robusticity metric

**DOI:** 10.12688/f1000research.139356.2

**Published:** 2024-07-10

**Authors:** James Fleming, Pia Merete Eriksen, Torsten Hugo Struck

**Affiliations:** 1 Natural History Museum, Universitetet i Oslo, Oslo, Oslo, 0562, Norway

**Keywords:** phylogenetics, bootstrapping, software

## Abstract

**Background:** The phylogenetic bootstrap, first proposed by Felsenstein in 1985, is a critically important statistical method in assessing the robusticity of phylogenetic datasets. Core to its concept was the use of pseudo sampling - assessing the data by generating new replicates derived from the initial dataset that was used to generate the phylogeny. In this way, phylogenetic support metrics could overcome the lack of perfect, infinite data. With infinite data, however, it is possible to sample smaller replicates directly from the data to obtain both the phylogeny and its statistical robusticity in the same analysis. Due to the growth of whole genome sequencing, the depth and breadth of our datasets have greatly expanded and are set to only expand further. With genome-scale datasets comprising thousands of genes, we can now obtain a proxy for infinite data. Accordingly, we can potentially abandon the notion of pseudo sampling and instead randomly sample small subsets of genes from the thousands of genes in our analyses.

**Methods:** We introduce Scoutknife, a jackknife-style subsampling implementation that generates 100 datasets by randomly sampling a small number of genes from an initial large-gene dataset to jointly establish both a phylogenetic hypothesis and assess its robusticity. We assess its effectiveness by using 18 previously published datasets and 100 simulation studies.

**Results:** We show that Scoutknife is conservative and informative as to conflicts and incongruence across the whole genome, without the need for subsampling based on traditional model selection criteria.

**Conclusions:** Scoutknife reliably achieves comparable results to selecting the best genes on both real and simulation datasets, while being resistant to the potential biases caused by selecting for model fit. As the amount of genome data grows, it becomes an even more exciting option to assess the robusticity of phylogenetic hypotheses.

## Introduction

The genomics revolution completely altered our understanding of phylogeny - the study of the relationships between organismal groups. By combining molecular and morphological data, our picture of the evolution of life has become clearer than ever before. ^1^^,^^2^ We are now in the process of entering the next phase of the genomics revolution, however, where beyond single or multi-gene datasets, researchers are now able to accurately sequence whole genomes from multiple species with relative ease. ^3^^–^^5^ This new era of “big data”, properly leveraged, promises to revolutionise our understanding of phylogenetics in the same way our prior understanding was revolutionised by the discovery of genomics itself. However, appropriately handling this new data is key to unlocking its potential. First, the robustness or statistical significance of these new results must be appropriately assessed. Second, assurances must be provided that the phylogenies reflect the actual biological processes and are not being misled by reconstructive biases. ^1^^,^^6^^,^^7^

**Figure 1.**
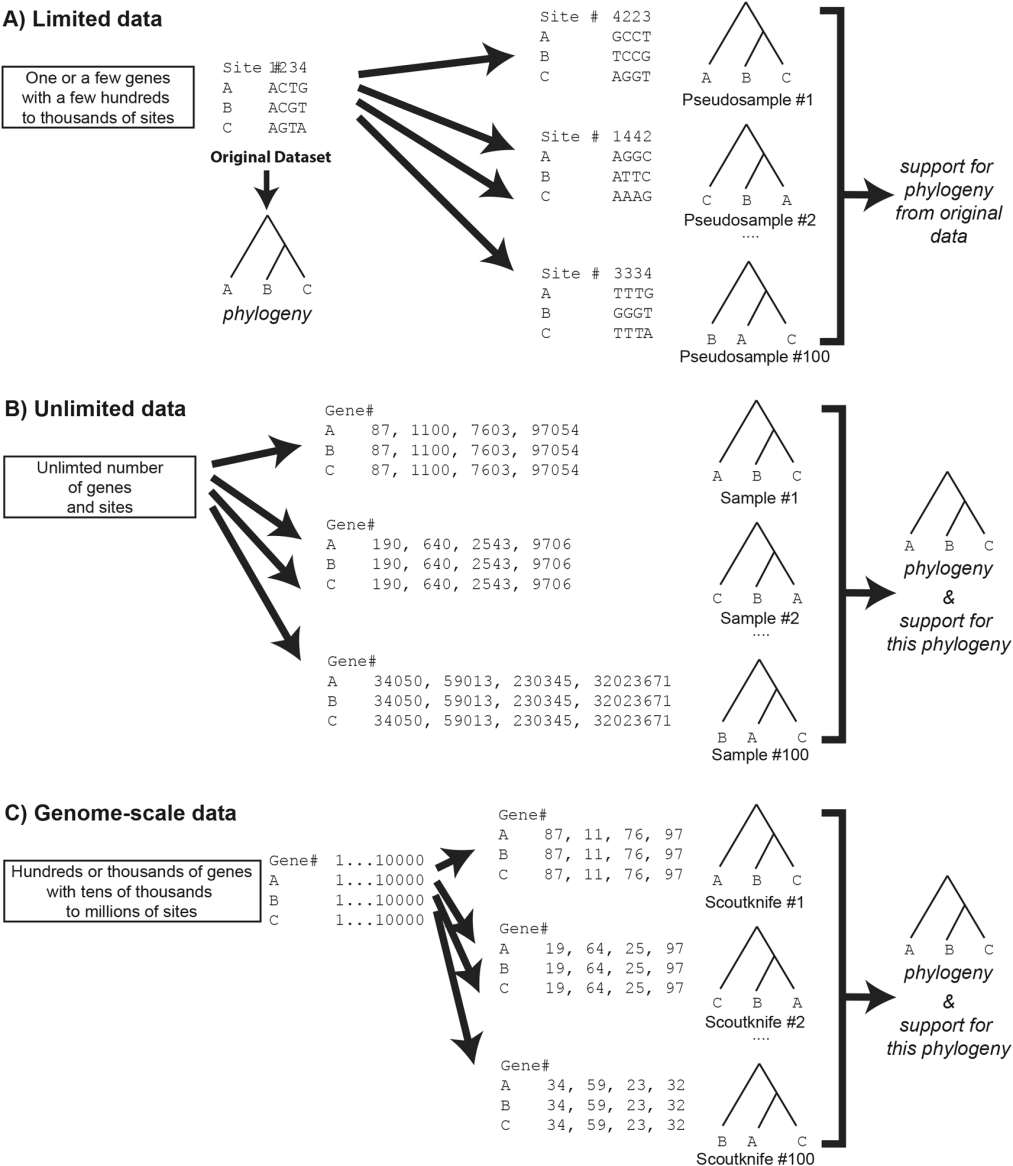
A figure showing a bootstrap pseudosampling process (Panel A) and a Scoutknife sampling process (Panel C), with the theoretical unlimited data jackknife sample in the middle (Panel B). Note that Scoutknife bears more similarity to unlimited data sampling than a traditional bootstrap. Scoutknife may not take the same gene twice within the same sample but may take the same gene multiple times between samples – see Scoutknife replicate #1 and #2, which both sample gene #97. The structure of this figure is based upon Hillis *et al.* (1996), Chapter 11, page 508, Figure 33. ^18^

The robustness and reliability of a phylogenetic topology and its branches can be quantified in a number of ways, such as through Bayesian posterior probabilities ^8^ or the Likelihood Ratio Test family of support values. ^9^^,^^10^ One of the most common, however, is the bootstrap support value. ^11^ A measure of statistical robustness, the bootstrap was first applied to phylogenetics by Felsenstein in 1985. In its implementation, the phylogeny is reconstructed from the limited source dataset and the bootstrap creates multiple pseudo-replicates of the source datasets – effectively multiplying the signal of some sites in the datasets and removing the signal of others ( Figure 1A). A variation of this approach is the generation of pseudo-samples by jackknifing – resampling only a fraction of the sites (e.g., 60% or 80%) from the source dataset. ^12^ This measures robustness, assessing how many of the sites in the source dataset support the final phylogeny – or more specifically its branches – and thereby whether there is a broader consensus for the proposed most likely topology – or for a particular branch – amongst the source dataset’s component sites. ^13^ These pseudo-replicates can be thought of as a measure of the evenness of signal across a phylogenetic dataset. In each pseudo-sample, certain sites are removed, while other sites remain (jackknifing) or are duplicated (bootstrapping), which has the effect of muting the removed sites whilst amplifying the signal of the other sites.

Bootstrapping is particularly useful where data is limited, such as in single or limited gene datasets, where pseudo-replicates can greatly proportionally increase the effective size, and thereby statistical power, of the analysis. ^11^ It was originally implemented as a surrogate for a robust statistical sampling procedure from theoretically unlimited data. ^14^ In the case of unlimited data, one could generate random samples from these data, then generate the tree of each sample and determine the overall phylogeny by including measurements of the robustness across all generated trees ( Figure 1B).

In the modern era, genome-scale data is being generated for a rapidly increasing number of species across the tree of life. ^4^^,^^15^^–^^17^ When data is plentiful, the reliance on pseudo-samples becomes less necessary. With thousands of genes and millions to billions of base pairs on the horizon for phylogenetic analyses, one can safely assume that the theoretical assumption of unlimited data is not violated. Accordingly, the data can be repeatedly sampled directly, trees reconstructed and the phylogeny and statistical support determined ( Figure 1C) as outlined directly from the aforementioned unlimited data. It is this assumption that forms the philosophical basis of the Scoutknife approach.

At the same time, however, the reconstruction of the species history can be challenging due to either methodological incongruence (i.e., not all genes contain information about the species history that we can correctly decipher) or biological incongruence (i.e., not all genes follow the species history). ^1^^,^^6^^,^^7^ Making use of large amounts of genome data comes with both an important caveat and an important boon: while methodologically incongruent genes can be removed by a number of tools to identify branch length heterogeneity, compositional heterogeneity and site saturation, genes that are excluded due to biological incongruence may contain real biological information that alters our understanding of species relationships. ^1^

Currently, phylogenetics has adopted a conservative stance, ^1^^,^^18^ selecting genes that fit well within our models of evolution ^19^^,^^20^ – this has the benefit of evading artifactual topologies, but it may also be presenting us with hypotheses of evolution that are preselected according to our own biases. As a result, this could cause us to incorrectly adopt great confidence in hypotheses that are not as well supported by the data as they first appear. ^21^ In this respect, an approach that is conservative towards the models may not be conservative towards confidence in our phylogenetic hypotheses. Scoutknife presents an alternative. ^11^^,^^22^ By randomly sampling data across the genome, the key hurdle for this methodology is assessing whether methodological incongruence significantly negatively influences the final hypothesis, or whether the false signal supplied by these model violations is outweighed by the addition of the real biological signal supplied by the sheer density of big data. If the latter is true, Scoutknife may represent a better way forward for generating phylogenetic topologies – one that is robust to methodological incongruence whilst expressing the biological incongruence that is present in the data.

Here we present Scoutknife, a new method for assessing topological support while simultaneously reconstructing phylogenetic relationships. In contrast to the traditional bootstrap and jackknifing approaches, Scoutknife discards the creation of artificial pseudo-replicates of sites to instead use large multi-gene inputs to create true replicate samples of a larger pool of genes. Through this procedure, Scoutknife is similar to gene jackknifing. Jackknifing of gene data has been historically used in two ways. First, to assess the effect of gene order on topological confidence in concatenated gene datasets. ^23^ Second, to reconstruct the phylogeny of a larger dataset by subsampling a random set of genes (or gene trees), ^24^ to assess the robustness of the obtained phylogeny, as in a traditional bootstrap approach. ^25^^–^^27^

Both jackknife and Scoutknife datasets are formed by reducing the overall number of genes in comparison to the original sample and the same gene will never occur twice in the same replicate. The first gene jackknifing procedure reduces the dataset via a subtractive process. ^23^ Jackknife in this form is a useful tool for understanding the impact of gene order on topologies - by otherwise keeping order constant, the effect of removal can be directly assessed. ^23^ In contrast, the second form for gene jackknifing and Scoutknife draw their samples randomly from the source dataset. The difference between gene jackknifing as applied nowadays ^25^^,^^26^ and Scoutknife lies primarily in the subtle difference in their philosophical basis. The second form of gene jackknifing is applied after the phylogenetic reconstruction based on the entire dataset and with the sole purpose of assessing the robustness of the results ( Figure 1C). In contrast, Scoutknife is a naive and unbiased way to measure support with genome-scale data, while reconstructing phylogenetic relationships. It does this by generating a sample number of datasets, each consisting of a user-specified number of randomly selected genes and forming a consensus tree from the results ( Figure 1).

Scoutknife can also be used to implement the *a posteriori* gene jackknifing procedure. To our knowledge, it is the first user-friendly implementation of this procedure. Scoutknife support values can be attached to nodes of a maximum likelihood tree, similar to conventional bootstrap or jackknife support.

Here we show, across 18 real and 100 simulation datasets, Scoutknife consensus trees produce comparable topological results to selecting the best genes within the dataset using GeneSortR, ^19^ and is robust to poor data occupancy. In addition, Scoutknife proves to be more granular in its assessment of topological reliability than traditional bootstrap values, allowing researchers to be more cautious and informed about their topological hypotheses than ever before.

### Approach

Scoutknife takes a “brute force” approach to assessing phylogenetic robustness, simply asking the question - “how robust is the most likely tree to topological signal across the entire dataset?”. Rather than generating pseudo-samples by randomly sampling sites, as in a traditional bootstrap, ^11^ Scoutknife generates real data samples by randomly sampling genes to create randomly assembled concatenated multi-gene datasets ( Figure 1C). In theory, though some of these genes contain low signal and others contain signal not consistent with the species phylogeny – either by methodological or biological incongruence ^1^^,^^7^ – the majority should contain at least some signal of the overall species tree, thereby allowing us to more robustly quantify not only the degree of support for a given taxonomic topology, but also the degree of discordance within the constituent genomes themselves. This naïve method may further allow us to resolve new phylogenetic hypotheses that have previously been neglected due to a focus on data selection.

First, the multi-gene dataset is divided into individual gene alignments. These alignments are then randomly selected to form 100 multi-gene partitioned datasets of a size equal to that selected by the user. The same gene cannot be selected twice by the same dataset ( Figure 1C) – a key difference from a traditional bootstrap ^11^ – but may appear multiple times across different datasets. Within our real dataset analyses, this sampling comprised 100 100-gene datasets selected from multi-gene datasets ranging from 1049 to 5105 genes ( Table 1). Our simulated datasets comprised 100 replicates of a 1049 gene dataset, from which 100 100-gene datasets were then sampled.

## Materials & Methods

### Dataset construction and analysis

For both real and simulation analyses (for details see below), 100 genes were randomly selected 100 times from the source datasets, generating 100 100-gene concatenated sample datasets using the Scoutknife Package ( https://github.com/JFFleming/Scoutknife). The Scoutknife script package requires catsequences ^28^ to be installed as a prerequisite, available at ( https://github.com/ChrisCreevey/catsequences).

Phylogenies for each Scoutknife dataset were constructed under IQ-Tree v1.6.12 ^29^ using ModelFinder, ^30^ with a separate model applied to each gene and no partition merging. As a data density-based technique, Scoutknife might be expected to perform better in high data density scenarios where partitions can be comfortably merged. As such, this was intended to limit the efficacy of Scoutknife further and test its performance under a scenario with more highly variable best fit models than might be expected under normal conditions, whilst conserving computational effort considering the large number of test datasets and simulations. To further facilitate parallelization of the analyses, the phylogenetic analyses of the datasets were submitted using Scoutknifette ( https://github.com/Togtja/scoutknifette). Scoutknifette is a custom high-performance computing (HPC) webhook for the group messaging service Discord ^31^ that can be easily modified for any HPC tasks that require multiple submission batches and queue tracking.

The trees produced by each Scoutknife sample dataset were then concatenated into a single treelist file (see Underlying Data in our Data Availability Statement), and a consensus tree was constructed using bpcomp, available in Phylobayes, ^32^ by using a burnin of 0 and a sampling rate of 1, sampling each tree in the treelist. Trees were constructed as both 70% strict consensus and 50% majority consensus trees, and the results were compared. In two cases (Araneae and Lepidoptera), 30% plurality consensus trees were constructed using the same method, to further explore the data, as explained in the results and discussion section. In a single case (Actinopterygii), the low occupancy of two species in particular ( *Muraenesox cinerus* with 1 gene and *Scomber scombrus* with 15 genes across the entire dataset of 1105) meant that many of the Scoutknife samples did not contain representatives from these taxa. To address this, we used sumtrees.py v 4.5.2, part of the DendroPy package, ^33^ as it is capable of building consensus trees from tree lists containing a variable number of taxa.

The Quartet Similarity, Quartet Divergence, Node Conflict, Node Agreement, Strict Joint Assertions, Semi-Strict Joint Assertions, Symmetric Difference, Marczewski-Steinhaus, Steel-Penny and Overall Similarity were measured with reference to the previously published topology of the 250 most informative genes of that dataset, as selected by GeneSortR. ^19^ In the case of the simulated datasets, the topology of the 250 most informative genes of the original source dataset, Milla *et al.,* (2020), as selected by GeneSortR, ^19^^,^^34^ was used. These similarity metrics were calculated using the ‘Quartet’ Library available in R. ^35^

### Real test datasets

To assess the efficacy of Scoutknife, we examined 18 real data datasets, ^25^^,^^34^^,^^36^^–^^51^ those used in a similar benchmarking study by GeneSortR. ^19^ These datasets range from 1049 to 5105 genes and from 30 to 332 taxa in size, comprising studies of animals, plants and fungi ( Table 1). In contrast to the prior study, genes with less than 50% occupancy were not removed: Scoutknife should show decreased performance at low occupancy levels, as it relies on data density, and so this should give a clearer picture of how the methodology performs across a variety of real datasets. The resultant tree topologies were then compared to the topology recovered by analysing the most informative 250 genes, as determined by GeneSortR, ^19^ to assess whether the same topological hypothesis was resolved by the Scoutknife Consensus Tree.

**Table 1.** A table listing the real data datasets used to benchmark the performance of Scoutknife. The first column names the taxa that form the ingroup of the phylogeny. The second names the original publication (although all source datasets are the same as those evaluated by Koch *et al.* (2021). ^19^ The third and fourth columns detail the number of taxa and the number of genes in the original alignment respectively.

Taxa	Study	Number of taxa	Number of genes
Actinopterygii	Hughes *et al.* (2018) ^36^	306	1105
Araneae	Fernández *et al.* (2018) ^37^	168	2365
Aspergillacea	Steenwyk *et al.* (2019) ^38^	93	1668
Blattodea	Evangelista *et al.* (2019) ^39^	66	3235
Echinoidea	Mongiardino Koch & Thompson (2021) ^40^	37	2356
Gnathostomata	Irisarri *et al.* (2017) ^25^	100	4593
Heliozelidae	Milla *et al.* (2020) ^34^	46	1049
Hemipteroids	Johnson *et al.* (2018) ^41^	193	2395
Hexapoda	Misof *et al.* (2014) ^42^	144	1478
Hymenoptera	Peters *et al.* (2017) ^43^	174	3256
Lepidoptera	Kawahara *et al.* (2019) ^44^	203	2098
Monilophytes	Shen, Jin *et al.* (2018) ^45^	73	2391
Myriapoda	Fernández *et al.* (2016) ^46^	51	2131
Opiliones	Fernández *et al.* (2017) ^47^	67	1550
Phasmatodea	Simon *et al.* (2019) ^48^	61	1097
Pseudoscorpiones	Benavides *et al.* (2019) ^49^	48	2473
Saccharomycotina	Shen, Opulente *et al.* (2018) ^50^	343	5105
Scorpiones	Sharma *et al.* (2018) ^51^	43	1464

### Simulation datasets

To further assess the efficacy of Scoutknife, we generated 100 simulation datasets using the Alignment Mimic function of AliSim, as implemented in IQTree v2.2.0. ^52^^,^^53^ For this, 100 simulations were independently created for each gene in the Milla *et al.,* (2020) ^34^ Heliozelidae dataset, as it represented a small-sized dataset of those within our real data study, at 1049 genes, and as such should have presented a challenge for Scoutknife. Furthermore, AliSim’s alignment mimic ^52^ allows us to generate alignment datasets that mimic real genes, complete with low occupancy and reasonable variations in alignment length. Alisim was implemented with the following command:

iqtree2–alisim<Output>−s<Gene>−−num−alignments100

For each set of 1049 simulated genes, 100 100-gene Scoutknife datasets were constructed, and then analysed using IQ Tree as with the real datasets. The Quartet Similarity, Quartet Divergence, Node Conflict, Node Agreement, Strict Joint Assertions, Semi-Strict Joint Assertions, Symmetric Difference, Marczewski-Steinhaus, Steel-Penny and Overall Similarity were then measured with reference to the previously published topology generated by analysing the 250 most informative genes of the Milla *et al.* (2020) dataset as selected by GeneSortR. ^19^ As each gene was simulated independently, it should in theory retain the topology of that initial single gene dataset, thereby replicating the discordance present in the original dataset. Furthermore, by directly comparing our random samples of simulated datasets to the most informative genes of the source dataset, this should disadvantage Scoutknife, as some of the simulated data may support a separate alternative topology to either the single gene or the real informative gene topology.

### Assessing the efficacy of Scoutknife

For each dataset, we calculated a variety of quartet-based similarity metrics: the Quartet Divergence, ^54^ the proportion of nodes that did not conflict between trees, the proportion of nodes that explicitly agreed between trees, the proportion of strict and semi-strict joint assertions, ^55^ the symmetric difference between trees ^56^ and the Steel-Penny ^57^ and Marczewski-Steinhaus similarity metrics. ^56^ Concordance with the initial study’s topology was first measured by assessing the proportion of nodes that explicitly agreed between topologies and then the proportion of nodes that did not conflict with the recovered topology. This could then be further scrutinized using the Quartet Divergence and then the Marczewski-Steinhaus (MS) measurement, which compares the distinctly resolved quartets in common between both trees. The remaining quartet measurements are present in our Underlying Data, available at DataDryad. Robinson-Foulds (RF) distances were not used due to Scoutknife’s propensity to recover nodes with conservatively low amounts of support. Polytomies are known to bias RF distances as they rely on a completely resolved tree, and this would be incompatible with the Scoutknife approach, which explicitly favours polytomies as representations of incongruent signal in the genome. ^58^

## Results & Discussion

### Real datasets

**Figure 2.**
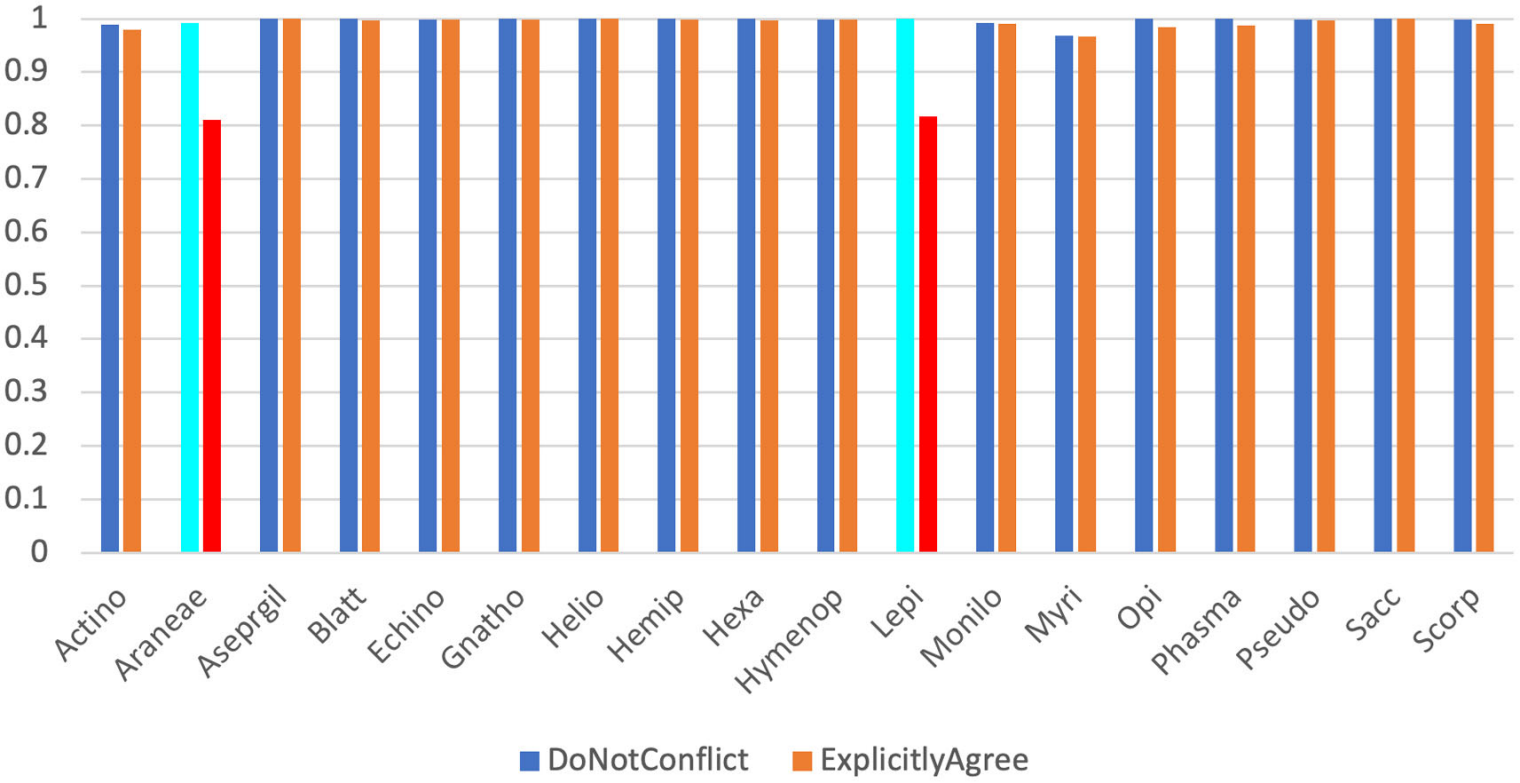
A dual bar chart showing proportion of non-conflicting nodes (in blue) and explicitly agreeing nodes (in orange) for each dataset. The two datasets discussed further in the text, Araneae and Lepidoptera, are highlighted in light blue (for non-conflict) and red (for explicit agreement) respectively.

**Figure 3.**
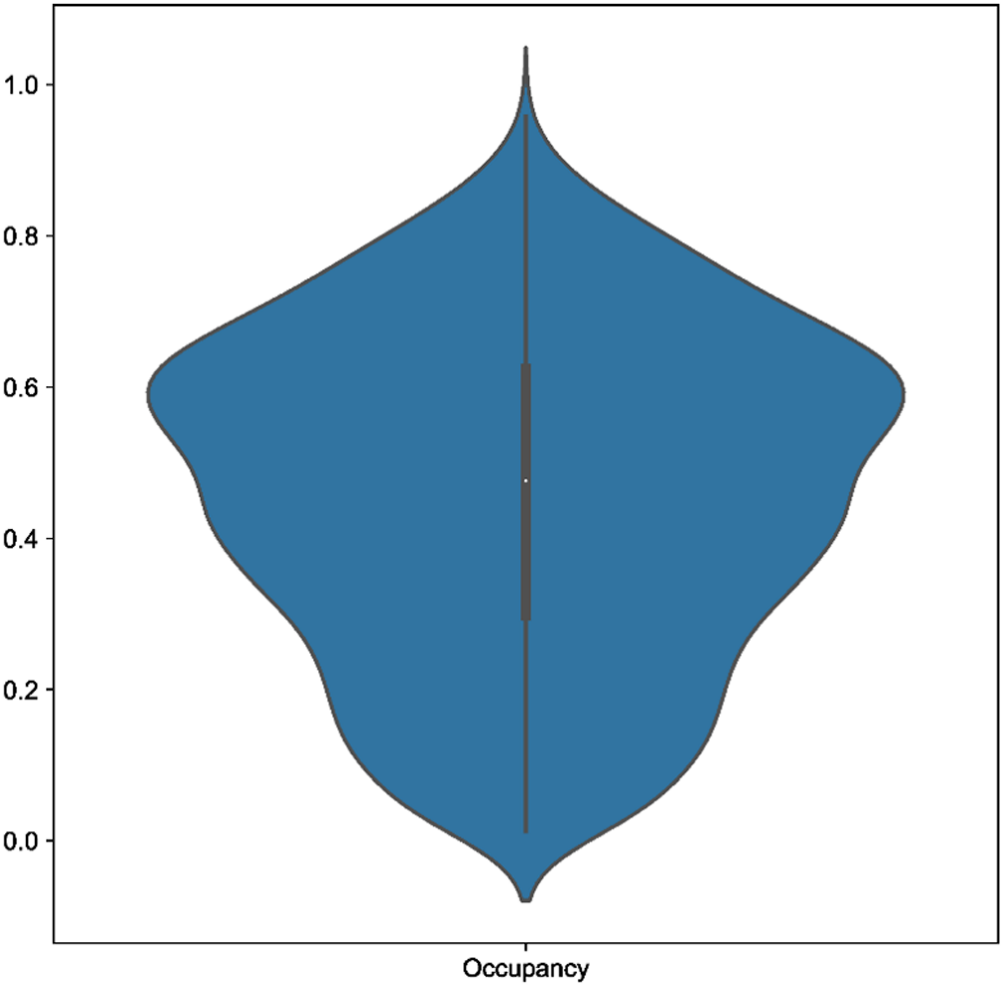
A violin plot showing the distribution of gene occupancy across the Araneae dataset by Fernández *et al.* (2018). ^31^ A large proportion of low occupancy genes may cause issues for Scoutknife resolution.

Across our 18 real test datasets, on a majority consensus tree, Scoutknife only struggles to recover the topology initially recovered by the original study in two cases (indicated by an explicit agreement of nodes below 90%, Quartet Divergence greater than 5% or a Marczewski-Steinhaus below 0.9) ( Figure 2). In the Araneae, Scoutknife achieved an “explicit agreement” value of 81.10%, Marczewski-Steinhaus of 0.80, and quartet divergence of 9.87%, which prompted us to further examine the dataset. The average occupancy of the dataset, when including genes with below 50% occupancy, is 46%. Furthermore, only 97 of the 2366 genes in the dataset had an occupancy greater than 80% ( Figure 3). In this case, it appears that Scoutknife struggles with lower resolution data, and that large amounts of missing data may be a genuine challenge to the efficacy of the method. However, when assessed using the more liberal criteria of measuring the proportion of nodes that do not conflict with the published tree (which is a measure that accounts for the uncertainty expressed by polytomies), 99.17% of recovered nodes were found to not be in explicit conflict ( Figure 2). This suggests that 18% of this discordance is caused by a conservative assessment of support in the data considering its low occupancy, not by disagreement in inference.

The second dataset that appeared to struggle under the Scoutknife approach was the Lepidoptera dataset. Here, only 81.66% of nodes were found to explicitly agree with the published topology, and it produced an MS value of 0.82 and quartet divergence of 9.19%. As in the Araneae, we find that 99.95% of the nodes did not conflict with the published topology. However, the reasons for this discordance within the Lepidoptera are less clear. This dataset had the sixth highest occupancy of the real datasets (88.8%), many of which produced more well-resolved Scoutknife consensus trees. Furthermore, GeneSortR measured the “Usefulness” of the dataset as the third highest of the selected study sets (0.33, on a range from 0.14-0.48). ^19^ Changing the minimum consensus value to produce a Scoutknife tree from a majority consensus tree to a 30% plurality support tree increases the Marczewski-Steinhaus value to 0.93, decreases quartet divergence to 3.63% and increases the number of nodes found to explicitly agree with the published topology to 92.83% (Underlying Data). This suggests that the discordance within the Lepidoptera dataset may be a true biological property of the history of the group, and that the difference between the Scoutknife result and prior published results may be indicative of gene selection and analysis methods strongly favouring one of a series of genuine alternative hypotheses that Scoutknife prefers to represent as a polytomy. This assertion is particularly supported when contrasted against the Araneae dataset – there, changing the minimum consensus value to produce a 30% plurality support tree increases the Marczewski-Steinhaus value from 0.80 to only 0.87 (0.07 increase Araneae vs. 0.11 in Lepidoptera), decreases quarter divergence from 9.87% to 6.77% (3.1% decrease vs 5.56% in Lepidoptera), and increases the number of nodes that “explicitly agree” from 81.10% to 87.59% (6.49% increase vs. 11.17% in Lepidoptera), a much smaller overall change in comparison.

In the opposite direction, on a stricter 70% consensus tree, Scoutknife achieves an average of 99.85% nodes not conflicting with the trees produced by GeneSortR, ranging from 100% to 99.16%. At this higher value, however, explicit agreement varies between 56.91% (in the Araneae) and 99.90%, with an average of 94.35% (or 96.56% if the Araneae are excluded). This is due to the innate conservatism of Scoutknife – as the consensus guideline is increased, it is more likely to favour collapsing more nodes into polytomies – the average decrease in Explicit Agreement with the GeneSortR tree between the majority consensus trees and the 0.7 consensus tree is 2.91%, with values ranging from 0% (Echinoidea) to 24.18% (Araneae).

### Simulation datasets

**Figure 4.**
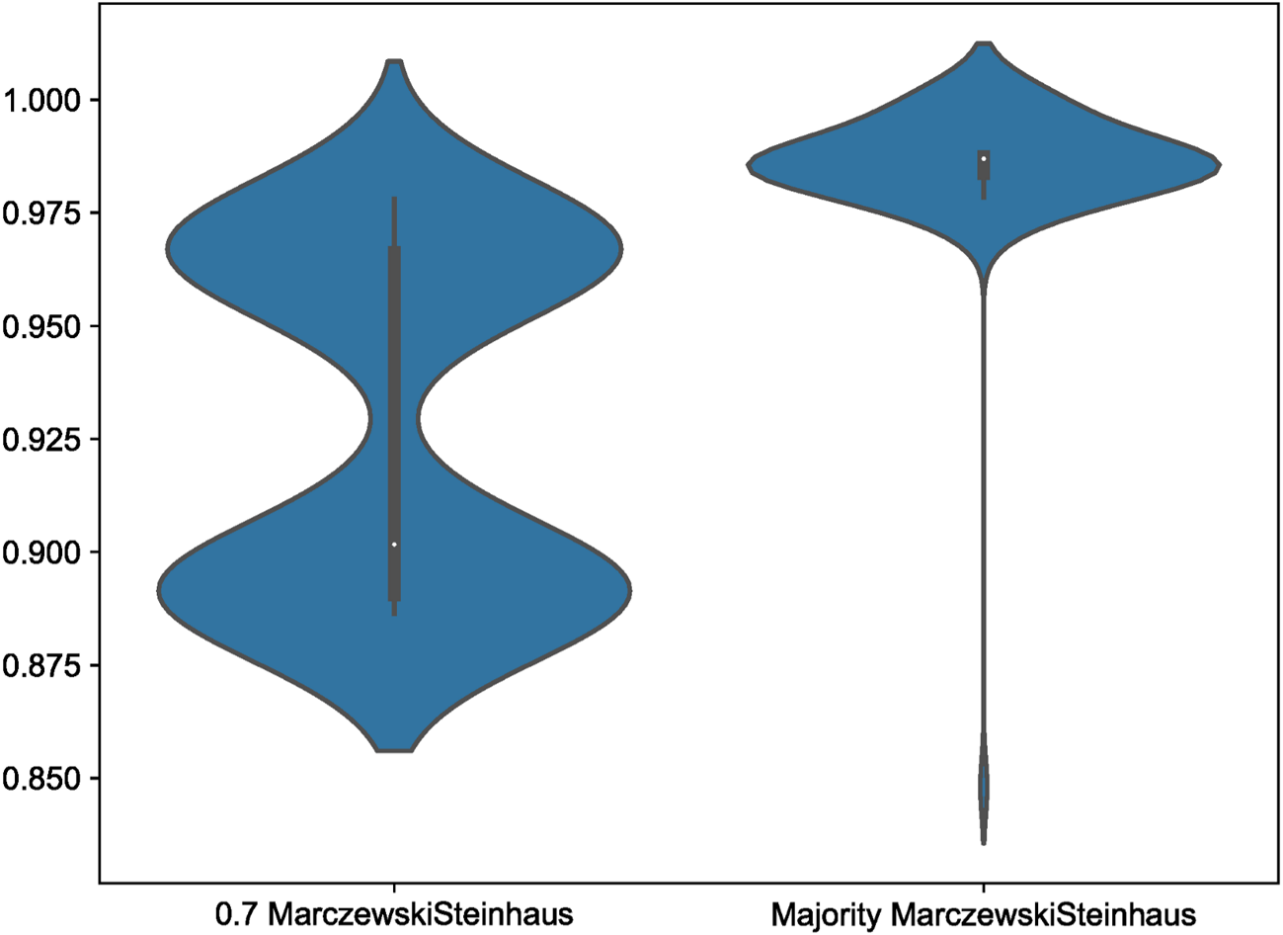
A violin plot showing the distribution of Marczewski-Steinhaus values between Scoutknife Consensus trees and the GeneSortR Most Informative 250 Genes Tree at both a 0.7 strict consensus and 0.5 majority consensus. Note the long tail on the Majority Marczewkski-Steinhaus violin, representing Simulation 20.

Within our simulation datasets, Scoutknife consistently recovers topologies that are consistent with the GeneSortR tree – the 70% strict consensus simulation trees recover no conflicting nodes with the GeneSortR topology. However, across the 100 simulation consensus trees, not all explicitly agree with the nodes resolved by GeneSortR ( Figure 4). At 70% strict consensus, explicit agreement varied between 97.81% and 88.64% with an average of 92.85%. This represents the greater conservatism of Scoutknife as a method – across all analyses, it prefers to resolve as polytomies, rather than bifurcations, representing the discordance across the genes in the dataset. This is further confirmed by the Marczewski-Steinhaus similarity index, which is consistent with the explicit agreement values (varying from 0.89 to 0.98 with an average of 0.93), suggesting that the only difference between the Scoutknife and GeneSortR topologies is in the existence of polytomies.

Examining the simple majority consensus trees, requiring a consensus of only 50% of resolved gene trees to resolve the node and not 70%, two bifurcating topologies were produced that conflicted with the GeneSortR topology (Simulation 20 and Simulation 98), reducing the average nodal “Do Not Conflict” value from 100% to 99.92%. While the Simulation 98 topology was very similar to the GeneSortR topology (Quartet Divergence 0.010), Simulation 20 showed significant divergence from the GeneSortR topology (Quarter Divergence 0.082).

The discordance in Simulation 20 is caused by a single node distinguishing the *Pseliastis* group, *Hoplophanes* group and the ( *Heliozela/Antispila/Antispilina/Holocacista/Coptodisca*) group. The Scoutknife analysis of Simulation 20 recovers this node with a support of 0.51 for ( *Pseliastis*+ *Hoplophanes*), the topology that is not favoured by GeneSortR or the remaining 99 Scoutknife simulations. GeneSortR recovered the alternative topology ( *Pseliastis*+ *Heliozela/Antispila/Antispilina/Holocacista/Coptodisca*) with a boostrap support of 83, the second least supported node in the entire Heliozelidae dataset, suggesting that there is considerable conflict at this node. The GeneSortR topology was also recovered by the Scoutknife analysis of the original dataset with a support of 0.62 and by the original study ^34^ with a UF bootstrap support of 65.1 and an SH-aLRT result of 72. Across our other Scoutknife simulations ( *Pseliastis*+ *Heliozela/Antispila/Antispilina/Holocacista/Coptodisca*), was recovered with support ranging from 0.57 to 0.86. As a particularly short branch in all analyses, this could suggest that Scoutknife struggles to discern the topology when fewer genes have the capacity to resolve a node, or when incomplete lineage sorting increases substantially due to short branch lengths.

Across all 100 Simulation datasets, when the consensus value was lowered to a majority consensus tree, explicit agreement with the GeneSortR topology increased from an average of 92.85% to 98.62%, with explicit agreement values varying from 91.19% to 100%. This shows that, on average, in 5.77% of the nodes in the tree where GeneSortR displayed high confidence, Scoutknife instead assigned these nodes between 50 and 69% support. In accordance with this, Marczewski-Steinhaus similarity scores increased from an average of 0.93 to 0.99, with a variance from 0.85 – the aforementioned Simulation 20 – to 1 ( Figure 4). Discounting the outlying Simulation 20, Marczewski-Steinhaus similarity scores vary from only 0.97 to 1. This further showcases the benefits of Scoutknife’s more conservative approach, making use of the diversity of data to give a more informed approximation of support from the gene trees without necessarily losing resolution at these key nodes.

### Scoutknife and per-taxon gene occupancy: A hypergeometric distribution

Our expectation was that as datasets became larger, they would become easier for Scoutknife to assess. However, instead, in both the Araneae and the simulation datasets, we found that Scoutknife was far more severely affected by per taxa dataset occupancy, rather than dataset size. In the simulation datasets, this takes the form of the simulation genes derived from *Nothofagus*, which is present in only 98 of the 1049 genes in each dataset. A consequence of this is that, in a truly representative 100-gene Scoutknife sample, a gene containing *Nothofagus* should be selected 9.3 times.

As an individual Scoutknife sample cannot select the same gene twice (although the same gene can be selected multiple times between samples), the probability of selecting any given gene can be modeled as a hypergeometric distribution. This presents us with an understanding that only 60.52% of 100-gene Scoutknife samples will comprise at least 9 *Nothofagus* genes. On the other hand, there is a 99.99% chance that a 100-gene dataset contains at least one *Nothofagus* gene among the one hundred. However, in a 50-gene Scoutknife sample, there would be a 0.66% chance that 0 genes containing this taxon would be selected across the 1049. That means that across 100 50-gene Scoutknife samples, 1 sample of the 100 is likely to contain no representation of this taxon.

In this way, taxa with low gene occupancy have a far more notable effect on Scoutknife than reducing the number of genes, which evenly reduces the number of genes for all taxa in the dataset. This is not surprising as the principal assumption of the ScoutKnife procedure ( Figure 1C) is that given genome-scale data works as a surrogate for unlimited data ( Figure 1B). Accordingly, the power of ScoutKnife is driven by the availability of genome-scale data across the entire dataset and not just parts of it. The taxa with the lowest genomic representation set the ceiling for Scoutknife’s effectiveness, rather than those with reference genomes. All the 18 datasets used for this study were compiled before the reference genome revolution, which is still very recent ^16^^,^^17^ and still restricted to only certain sections of the tree of life. Hence, for many taxa, genome-scale data at EBP minimum standards ^59^ are still lacking. However, in the near future, the full potential of ScoutKnife can be brought to bear on these data. Our analyses already strongly indicate the potential of these methods in comparison to others through their conservativism in tree resolution and support values due to a higher susceptibility to the biological and methodological incongruence in the data.

In the meantime, the reduced power of ScoutKnife due to taxa with reduced genomic representation can be addressed by increasing the number of genes selected by a Scoutknife sample relative to poor taxon occupancy. This increases the absolute number of genes containing the low occupancy taxa in the dataset, though it will not affect the proportional representation of the low occupancy taxa. For example, to consider the *Nothofagus* earlier, a 200-gene Scoutknife dataset would increase the chance of observing 9 *Nothofagus* genes in any given Scoutknife sample from 60.52% to 99.84%. By doubling the size of the Scoutknife sample, a representative number of genes would be 18. However, simply increasing the raw representation of genes may aid Scoutknife resolution. This approach deviates from the naïve sampling strategy and introduces missingness as a selection parameter. On the other hand, this is often already done explicitly or implicitly as some genes can only be found in certain ingroups, for example, due to a gene duplication event, and so are generally excluded from these analyses in the dataset compilation step.

Among the tools available at the Scoutknife Github is a Hypergeometric distribution calculator designed with Scoutknife in mind, to help researchers understand the composition of their Scoutknife samples prior to analysis. In addition, the Scoutknife “auto” function uses this distribution to calculate how many genes must be sampled across the input dataset in order to ensure a 99.9% chance of any given Scoutknife dataset containing that gene, and then automatically formats a Scoutknife analysis for the user based on these parameters.

## Conclusions

Selection-based metrics have rightly dominated phylogenetic discussions for a great number of years, but in the era of big data, transitioning towards methods that make best use of the increased analytical power of whole genomes may be more prudent. Our results, and the Scoutknife methodology, show that, contrary to accepted wisdom, model violations and incongruence can be overcome by sheer density of data. What results is a more neutral look at phylogenetic relationships, rather than one biased by our own notions of what makes genes suitable for phylogenetics. A helpful side effect of this is an increase in computational efficiency: rather than assessing individual gene trees prior to multi-gene analysis, 100 smaller Scoutknife datasets assess the robustness of a total dataset analysis or form the basis of a consensus tree. In many cases across our datasets, Scoutknife appears to recover the same relationships as before, but is also able to quantify our confidence in hypotheses of shared evolution efficiently and conservatively. In the future, this may be critical to a more holistic view of phylogeny. In addition, as models improve, and model incongruence becomes less and less of a concern, as a model-neutral methodology, Scoutknife’s ability to assess true biological incongruence will only improve, making it not only an exciting option for the present, but an even more effective one in the future.

## Data Availability

• Source Data sets, ^25^^,^^34^^,^^36^^–^^51^ and their analysis within GeneSortR ^19^ were used to assess the efficacy of Scoutknife. All files used to assess the efficacy of Scoutknife can be found reproduced in our underlying data link (below). Further information on the Source datasets can also be found in the supplemental data for Koch *et al.* (2021). ^19^ • Both our real and simulated data analyses are available at DataDryad, along with copies of individual gene fasta files from Source Data sets ^25^^,^^34^^,^^36^^–^^51^ and the 250 most informative gene trees from Koch *et al.* (2021) ^19^ that were used to benchmark Scoutknife’s performance ( https://datadryad.org/stash/dataset/doi:10.5061/dryad.sxksn0383).

## Software availability

• Scoutknife is available at GitHub https://github.com/JFFleming/Scoutknife.
• Archived Scoutknife code is available at: 10.5281/zenodo.8160834

Data is available under the terms of a GNU General Public License v2.0 only
