# Supplementary material for: Scoutknife: A naïve, whole genome informed phylogenetic robusticity metric
Source: F1000Res. 2024 Jul 10;12:945. Originally published 2023 Aug 7. [Version 2] doi: 10.12688/f1000research.139356.2 (PMC11128044; doi:10.12688/f1000research.139356.2)
Supplement: Peer review history

# Reviewer report: Reviewer response for version 2

**DOI:** 10.5256/f1000research.168838.r301251

**Published:** 2024-07-18

**Authors:** Xianzhao Kan

**Affiliations:** 1 Anhui Normal University, Wuhu, China

In the revised manuscript, the authors have made substantial modifications as requested. As a reviewer, I am satisfied and recommend it for approval.

Is the work clearly and accurately presented and does it cite the current literature?

Yes

If applicable, is the statistical analysis and its interpretation appropriate?

Yes

Are all the source data underlying the results available to ensure full reproducibility?

Yes

Is the study design appropriate and is the work technically sound?

Yes

Are the conclusions drawn adequately supported by the results?

Yes

Are sufficient details of methods and analysis provided to allow replication by others?

Partly

Reviewer Expertise:

Bioinformatics, Genomics, and Molecular evolution.

I confirm that I have read this submission and believe that I have an appropriate level of expertise to confirm that it is of an acceptable scientific standard.

# Reviewer report: Reviewer response for version 2

**DOI:** 10.5256/f1000research.168838.r301250

**Published:** 2024-07-11

**Authors:** Paul Zaharias

**Affiliations:** 1 Institut de Systématique, Evolution, Biodiversité (ISYEB UMR7205 – CNRS), Museum National d'Histoire Naturelle, Paris, Île-de-France, France

I am satisfied with this version and have no further comments to make.

 Thanks to the authors and editorial team!

Is the work clearly and accurately presented and does it cite the current literature?

Partly

If applicable, is the statistical analysis and its interpretation appropriate?

Yes

Are all the source data underlying the results available to ensure full reproducibility?

Yes

Is the study design appropriate and is the work technically sound?

Yes

Are the conclusions drawn adequately supported by the results?

Yes

Are sufficient details of methods and analysis provided to allow replication by others?

Yes

Reviewer Expertise:

Phylogenetics, systematics and bioinformatics

I confirm that I have read this submission and believe that I have an appropriate level of expertise to confirm that it is of an acceptable scientific standard.

# Reviewer report: Reviewer response for version 1

**DOI:** 10.5256/f1000research.152626.r195414

**Published:** 2024-05-25

**Authors:** Anthony K. Redmond

**Affiliations:** 1 Smurfit Institute of Genetics, Trinity College Dublin, Dublin, Ireland

This well written and presented study by Fleming and colleagues provides an important and much needed alternative measure of robustness for phylogenetic inference based on gene-wise jackknife resampling. 

 The logical and practical ideas behind, and advantages of using, their approach are clearly explained and exemplified in comparison to the traditional site-wise bootstrapping approach.

 While I must admit that the study has not entirely convinced me that 'model violations and incongruence can be overcome by sheer density of data' (and this may be a bias of my own), I am nonetheless very pleased with the conservative and nuanced results provided by the new Scoutknife approach as compared to standard bootstrap. I believe the Scoutknife approach has great potential within the field and am happy to recommend the manuscript without revision. I am excited to see how the approach holds up to extreme lineage-specific violations on the modelling and orthology fronts.

 As a very minor point and optional point, I believe there may be additional studies worthy of discussion here that support some of the arguements made in the text. For example, Simmons et al. 2019 [ ref 1 ] previously proposed the use of gene-wise bootstrap for summary coalescent species tree methods, while gene wise bootstrapping has been used in other studies in the past (e.g. Simion et al 2017 [ ref 2 ]), and off the top of my head has been suggested to be conservative in at least Philippe et al 2019 [ ref 3 ].

Is the work clearly and accurately presented and does it cite the current literature?

Yes

If applicable, is the statistical analysis and its interpretation appropriate?

Yes

Are all the source data underlying the results available to ensure full reproducibility?

Yes

Is the study design appropriate and is the work technically sound?

Yes

Are the conclusions drawn adequately supported by the results?

Yes

Are sufficient details of methods and analysis provided to allow replication by others?

Yes

Reviewer Expertise:

Phylogenomics, Genome Evolution, Gene and Genome Duplication, Immunology

I confirm that I have read this submission and believe that I have an appropriate level of expertise to confirm that it is of an acceptable scientific standard.

## References

[1] : Gene-wise resampling outperforms site-wise resampling in phylogenetic coalescence analyses. *Mol Phylogenet Evol* .2019;131: 10.1016/j.ympev.2018.10.001 80-92 10.1016/j.ympev.2018.10.001 30391518

[2] : A Large and Consistent Phylogenomic Dataset Supports Sponges as the Sister Group to All Other Animals. *Curr Biol* .2017;27(7) : 10.1016/j.cub.2017.02.031 958-967 10.1016/j.cub.2017.02.031 28318975

[3] : Mitigating Anticipated Effects of Systematic Errors Supports Sister-Group Relationship between Xenacoelomorpha and Ambulacraria. *Curr Biol* .2019;29(11) : 10.1016/j.cub.2019.04.009 1818-1826.e6 10.1016/j.cub.2019.04.009 31104936

# Reviewer report: Reviewer response for version 1

**DOI:** 10.5256/f1000research.152626.r261535

**Published:** 2024-05-06

**Authors:** Xianzhao Kan

**Affiliations:** 1 Anhui Normal University, Wuhu, China

The manuscript, "Scoutknife: A Naïve, Whole-Genome Informed Phylogenetic Robustness Metric", introduces a novel tool, Scoutknife. In this method, the authors can potentially abandon the notion of pseudo-sampling and instead randomly sample small subsets of genes from the thousands of genes in their analyses. Furthermore, the authors validated the effectiveness of Scoutknife by applying it to 18 previously published datasets and 100 simulation studies. I believe that the development of this method and software will facilitate deeper research in phylogenetics.

 The following weaknesses should be addressed:

 1. In the Abstract section, the term "robusticity" is used frequently, but I think it is not a standard statistical term. "Robustness" would be more suitable.

  2. In the Conclusions of the Abstract, "phylogenetic hypotheses" should be changed to "phylogenetic hypothesis" because it refers to a singular hypothesis.

  3. In the paragraph 4 of Introduction, the sentence "Currently, phylogenetics has adopted a conservative stance, selecting genes that fit well within our models of evolution – this has the benefit of evading artifactual topologies, but may well be presenting us with hypotheses of evolution that are preselected according to our own biases, or incorrectly causing us to adopt great confidence in hypotheses that are not as well supported by the data as it first appears." is quite long and could be split into multiple sentences for clarity.

  4. In the paragraph 6 of Introduction, the phrase "true replicate samples" may be confusing. It would be helpful to clarify the difference between "true replicates" and "artificial pseudo-replicates".

 5. In the section on Scoutknife and per-taxon gene occupancy, the following sentence is unclear: "genome-scale data works as a surrogate for unlimited data". The authors should clarify the relationship between genome-scale data and the concept of unlimited data.

Is the work clearly and accurately presented and does it cite the current literature?

Yes

If applicable, is the statistical analysis and its interpretation appropriate?

Yes

Are all the source data underlying the results available to ensure full reproducibility?

Yes

Is the study design appropriate and is the work technically sound?

Yes

Are the conclusions drawn adequately supported by the results?

Yes

Are sufficient details of methods and analysis provided to allow replication by others?

Partly

Reviewer Expertise:

Bioinformatics, Genomics, and Molecular evolution.

I confirm that I have read this submission and believe that I have an appropriate level of expertise to confirm that it is of an acceptable scientific standard, however I have significant reservations, as outlined above.

# Response

**Published:** 2024-07-04

**Authors:** James Fleming

**Affiliations:** Naturhistorisk Museet, Universitetet i Oslo, Oslo, Oslo, Norway

We thank the reviewer for their consideration of our manuscript, and have attempted to clarify and address our text. We would like to especially thank them for point out the lack of clarity in points 4 and 5: these are quite critical concepts to understanding the wider manuscript, so it is important they are explained as clearly as possible.

 In order to address this, we have greatly revised the initial introduction section in order to hopefully explain the concepts and reasoning behind Scoutknife more clearly.

# Reviewer report: Reviewer response for version 1

**DOI:** 10.5256/f1000research.152626.r227172

**Published:** 2023-12-29

**Authors:** Paul Zaharias

**Affiliations:** 1 Institut de Systématique, Evolution, Biodiversité (ISYEB UMR7205 – CNRS), Museum National d'Histoire Naturelle, Paris, Île-de-France, France

This article introduces “Scoutknife”, a software designed for gene jackknifing in phylogenomic datasets. The authors evaluate their approach with numerous empirical and simulated datasets and using a range of similarity metrics. They conclude that Scoutknife is a reliable estimator of the robustness of phylogenetic hypotheses.

 I find the article well-written and easy to follow, and that “Scoutknife” could be a useful user-friendly software for phylogenomists. I only have two major comments, followed by some minor comments hereafter.

 First, the method is not new (contrarily to what can be read in the Introduction, paragraph 6); the procedure of gene jackknifing exists since at least 15 years ago [1,2]. As such, I would like to see in the Introduction a paragraph which would be a summary of the literature on the use of gene jackknifing, its assessed qualities, and drawbacks. This is however, the first time (to my knowledge) that a user-friendly software is suggested, and that there is a proper benchmarking of it, and the authors should emphasize on those aspects.

 Second, as the author point out in the discussion, one drawback of gene jackknifing (as it is with site-based jackknifing) is the problem of poor taxon occupancy, unfortunately a common problem in phylogenomic datasets. To address that, the authors wrote a “Hypergeometric distribution calculator” script to help the users better understand the taxon occupancy composition prior to analysis. I think this script should be automated and included as an option in the main “Scoutknife” approach as follow:

- The Scoutknife script automatically detects the number of genes in the folder and also automatically detects how many of those genes contain the least represented taxa.
- Then, Scoutknife calculates how many genes should be sampled at least so that there is >99.9% chances that the least represented taxon appears in at least one gene. This value could be considered the default minimal value of gene sampling for the user to guarantee the presence of all taxa in the subsets.

Making the use of this “Taxon Probability Calculator" more visible and user-friendly would be a great plus and a great input to the more arbitrary “100 genes” strategy fixed by the authors and would show an adaptiveness of the approach to each dataset.

 Some minor comments:

 Everywhere: “Pseudosampling” in Fig.1 caption is not spelled the same way as other places in the text (“Pseudo sampling”). Might I suggest as well to add a hyphen after all “pseudo” as in “pseudo-sampling”, “pseudo-samples” and “pseudo-replicates”?

 Discussion, penultimate paragraph: please expand EBP to Earth BioGenome Project

 Last comment, which is more of a curiosity : is there any particular reason you chose the synonym "robusticity' instead of the more traditional "robustness" present in the literature ?

Is the work clearly and accurately presented and does it cite the current literature?

Partly

If applicable, is the statistical analysis and its interpretation appropriate?

Yes

Are all the source data underlying the results available to ensure full reproducibility?

Yes

Is the study design appropriate and is the work technically sound?

Yes

Are the conclusions drawn adequately supported by the results?

Yes

Are sufficient details of methods and analysis provided to allow replication by others?

Yes

Reviewer Expertise:

Phylogenetics, systematics and bioinformatics

I confirm that I have read this submission and believe that I have an appropriate level of expertise to confirm that it is of an acceptable scientific standard, however I have significant reservations, as outlined above.

## References

[1] : Additional molecular support for the new chordate phylogeny. *Genesis* .2008;46(11) : 10.1002/dvg.20450 592-604 10.1002/dvg.20450 19003928

[2] : Phylotranscriptomic consolidation of the jawed vertebrate timetree. *Nat Ecol Evol* .2017;1(9) : 10.1038/s41559-017-0240-5 1370-1378 10.1038/s41559-017-0240-5 28890940 PMC5584656

# Response

**Published:** 2024-07-04

**Authors:** James Fleming

**Affiliations:** Naturhistorisk Museet, Universitetet i Oslo, Oslo, Oslo, Norway

We would like to thank the reviewer for their positive response to the paper, and we found the comments below incredibly useful in improving the manuscript (which we are currently uploading). To address their concerns one-by-one below:

 ==================

 First, the method is not new (contrarily to what can be read in the Introduction, paragraph 6); the procedure of gene jackknifing exists since at least 15 years ago [1,2]. As such, I would like to see in the Introduction a paragraph which would be a summary of the literature on the use of gene jackknifing, its assessed qualities, and drawbacks. This is however, the first time (to my knowledge) that a user-friendly software is suggested, and that there is a proper benchmarking of it, and the authors should emphasize on those aspects.

 ----- Scoutknife is not quite the same as gene jacknifing. The two are similar but quite distinct in their mathematical underpinnings and philosophical approach - in particular, jackknifing as typically implemented preserves the relative order of genes in the genome, and is also used predominantly to assess the effect of order on the resultant dataset, following initial phylogenetic tree construction. Scoutknife is much more similar to a classical bootstrapping approach, where the creation of alignment sets does not respect gene order. However, the reviewer is totally correct that these are relatively minor distinctions between the two approaches that were not clearly delineated in our initial manuscript, and that leaving out discussion of gene jacknifing is an important oversight. We have revised the initial introduction section to discuss jacknifing, and the differences between scoutknife and jacknife in more detail. We have also been more computationally explicit about the intentions of Scoutknife, with a new script, Scoutknife_Consensus.pl, being included in the GitHub, to allow users to easily generate consensus trees within the package itself, rather than relying on alternative programs as we have done in the manuscript itself.

 ====================

 Second, as the author point out in the discussion, one drawback of gene jackknifing (as it is with site-based jackknifing) is the problem of poor taxon occupancy, unfortunately a common problem in phylogenomic datasets. To address that, the authors wrote a “Hypergeometric distribution calculator” script to help the users better understand the taxon occupancy composition prior to analysis. I think this script should be automated and included as an option in the main “Scoutknife” approach as follow:

- The Scoutknife script automatically detects the number of genes in the folder and also automatically detects how many of those genes contain the least represented taxa.
- Then, Scoutknife calculates how many genes should be sampled at least so that there is >99.9% chances that the least represented taxon appears in at least one gene. This value could be considered the default minimal value of gene sampling for the user to guarantee the presence of all taxa in the subsets.

Making the use of this “Taxon Probability Calculator" more visible and user-friendly would be a great plus and a great input to the more arbitrary “100 genes” strategy fixed by the authors and would show an adaptiveness of the approach to each dataset.

 ----- That is a great idea! We have included an "--auto" flag inside Scoutknife that works as the reviewer describes.

 ======================

 Some minor comments:

 ------ We have made revisions based on all minor comments. For the reviewer's curiosity, it was mostly the first author writing naturally - I'm not sure I'd put too much thought into that particular terminology usage, but you are totally correct to point it out. I have adjusted the terminology there appropriately.
